# Papanicolaou tests and molecular analyses using new fluid-based specimen collection technology in 3000 Japanese women

**DOI:** 10.1038/sj.bjc.6601023

**Published:** 2003-06-10

**Authors:** N Masumoto, T Fujii, M Ishikawa, M Mukai, M Saito, T Iwata, T Fukuchi, K Kubushiro, K Tsukazaki, S Nozawa

**Affiliations:** 1 Department of Obstetrics and Gynecology, Keio University School of Medicine, 35 Shinanomachi, Shinjuku-ku, Tokyo 160-8582, Japan; 2 Department of Pathology, Keio University School of Medicine, 35 Shinanomachi, Shinjuku-ku, Tokyo 160-8582, Japan

**Keywords:** fluid-based specimen, cytological techniques, Hybrid Capture II, human papillomavirus, polymerase chain reaction

## Abstract

A fluid-based Papanicolaou test has been established to improve sample collection and preparation. This study was the first large-scale investigation in Japan to examine the feasibility of using fluid-based Papanicolaou specimens to detect human papillomavirus (HPV) using Hybrid Capture II and polymerase chain reaction (PCR). Three thousand patients who visited Keio University Hospital between October 2000 and February 2001 were enrolled in the study. The results of the fluid-based Papanicolaou tests corresponded well with those of conventional Papanicolaou smears (96.8% concordance). The sensitivities of cervical neoplasia detection using the fluid-based Papanicolaou test (73.9%) and Hybrid Capture II (76.3%, *P*=0.55) were not significantly different. Among the cervical intraepithelial neoplasia 3 and squamous cell carcinoma specimens, HPV 16 and HPV 52 were predominantly detected using the PCR method. Although some DNA samples extracted from the fluid-based specimens were degradaded, PCR and direct sequencing could be performed without difficulty even after 1 year of specimen storage. We conclude that fluid-based Papanicolaou specimens can be applied to investigate HPV infection.

The fluid-based Papanicolaou specimen collection method is widely used for primary cervical cancer screening (Awen *et al*, 1994; Roberts *et al*, 1997; Bolick and Hellman, 1998), largely because of the improvement in specimen quality, the advantage of increased sensitivity, and a reduction in the false negative rate for squamous intraepithelial lesions (Linder and Zahniser, 1997; Park *et al*, 2001), compared to the conventional Papanicolaou smear method. Fluid-based specimens can be stored at ambient temperature for a longer period; therefore, this collection system offers the advantage that once the specimen has been collected, there is no need to collect additional specimens from the patient to perform a second Papanicolaou test or conduct further investigations. Studies evaluating the clinical utility of Hybrid Capture II (HC II) human papillomavirus (HPV)-DNA testing have also been performed (Clavel *et al*, 1998; Lin CT *et al*, 2000a; Yamazaki *et al*, 2001; Castle *et al*, 2002). Genital HPV has been reported to be related to cervical cancer carcinogenesis and some types of HPV; HPV 16, for example, is associated with a high risk of cervical neoplasia (Josefsson *et al*, 2000; Woodman *et al*, 2001). HC II is a HPV detection test designed to detect 18 types of HPV using microtitre plates and is an appropriate method for HPV screening. We performed the HC II test using fluid-based specimens from patients in whom biopsy studies were also performed to determine whether this method is appropriate for detecting cervical neoplasia in Japan. We also performed HPV typing using fluid-based specimens. We previously reported an HPV-DNA transcript detection method using cytologic specimens and reverse transcriptase-nested polymerase chain reactions (PCR) (Fujii *et al*, 1995), and a method for detecting multiplex HPV infection using PCR single-stranded DNA-conformational polymorphism analysis (Nakagawa *et al*, 2002). We applied these methods to the fluid-based specimens and then performed direct sequencing of the PCR products.

HPV testing with PCR using fluid-based specimens, in conjunction with cytologic and biopsy follow-up, has been reported to be useful for estimating the significance of atypical squamous cells of undetermined significance (Crum *et al*, 1999), and the concordance rate of HC II and PCR has been reported to be approximately 90% for fluid-based specimens (Peyton *et al*, 1998). HPV screening with HC II and HPV-typing analysis can be performed using residual specimens without the need to collect a second specimen from the patient; this collection system is thus of great advantage to both patients and clinicians. To our knowledge, however, the feasibility of using the fluid-based Papanicolaou test in conjunction with HPV testing has not been examined in Japan. Japan has more than 5000 cytotechnologists, and cytology has been established as an independent method of screening for cervical neoplasia. This study is the first large-scale investigation in Japan to examine the utility of fluid-based Papanicolaou specimens. In addition, the storage conditions for fluid-based specimens are controversial, and some of the genomic DNA was degraded in the samples that we examined. Therefore, we also investigated the quality of the genomic DNA in the specimens. This study was undertaken to evaluate the feasibility of using fluid-based Papanicolaou specimens to detect HPV using both the HC II and PCR methods in Japan.

## MATERIALS AND METHODS

### Sample preparation

Three thousand patients who visited our clinic between October 2000 and February 2001 were enrolled in the study. The patient population consisted of a mixture of asymptomatic women and those who were being followed-up for previous atypical smears or the treatment of previous genital malignancies. Cervical samples were collected using both the conventional method and the ThinPrep method, simultaneously. The conventional smears were collected with a survexbrush and fixed in ethanol at the time of collection. The ThinPrep samples were collected using the ThinPrep Cytology Collection System (Cytyc Corporation, Boxborough, USA), according to the manufacturer's directions. Out of the 3000 cases, colposcopy and biopsy studies were performed in 557 patients. The ThinPrep sample vials were stored at ambient temperature and used for the HPV-DNA analysis within 12 months of collection. Specimens from 477 random patients in whom biopsy studies had also been performed were subjected to HPV-DNA detection using HC II (Digene Inc., Silver Spring, USA), while specimens from 146 random cases diagnosed as having cervical neoplasia on the basis of biopsy studies were subjected to HPV-DNA detection using PCR. The HC II and PCR test results were compared in 133 cases. HPV typing and sequencing analysis were performed in all the PCR-positive cases. Informed consent was obtained from each biopsy study participant after an oral explanation of the study.

### Fluid-based cervical cytology

Thin-layer slides were prepared using the ThinPrep 2000 Automated Slide Processor (Cytyc Corp., Boxborough, USA), according to the manufacturer's instructions. Briefly, the vial containing the cells was placed in the processor, and a dispersion cycle homogenised the cell suspension. The cells were then automatically collected on a polycarbonate filter membrane. A thin, evenly dispersed monolayer of cells was deposited from the filter onto the slide in a circle with a diameter of 20 mm. Extraneous mucus and blood were removed in the process. The slides were then manually removed from the processor and stained using the Papanicolaou method. All slides, both conventional and ThinPrep, were screened by three cytotechnologists (MS, HT, and YN). The diagnostic adequacy of the fluid-based Papanicolaou specimens was compared with those of both conventional Papanicolaou smear and biopsy specimens.

### HPV detection using HC II

HPV-DNA detection was performed using the commercially available HC II hybrid capture technique. Fluid-based specimens were analysed for the presence of low-risk HPV types 6, 11, 42, 43, and 44 using a Probe A cocktail, and for high-risk HPV types 16, 18, 31, 33, 35, 39, 45, 51, 52, 56, 58, 59, and 68 using a Probe B cocktail. The sensitivity of the HC II method for detecting cervical neoplasia was then compared with that of the ThinPrep slides. The enzyme-linked immunosorbent assay was based on a sandwich hybridisation followed by a nonradioactive alkaline phosphatase reaction with chemiluminescence on microplates.

### DNA extraction

Approximately 10 ml of preserved fluid was centrifuged at 3000 r.p.m. for 30 min. The pellet was washed once in phosphate-buffered saline, followed by genomic DNA extraction using proteinase K and phenol–chloroform treatment. The quality and quantity of the extracted genomic DNA were monitored using ethidium bromide-stained agarose gel electrophoresis.

### Polymerase chain reaction

The presence of intracellular HPV DNA was determined using PCR analysis with consensus primer pairs (L1C1, L1C2) (Yoshikawa *et al*, 1991), designed to amplify an approximately 250-bp segment of DNA. These consensus primer pairs target the HPV L1 open reading frame and detect a broad range of genital HPVs. A 50 *μ*l volume containing 20 mM Tris HCl buffer (pH 8.0), 50 mM KCl, 0.2 mM dNTP mix, 2 mM MgCl2, 0.5 *μ*M of each forward and reverse primer, and 0.25 U of *Taq* polymerase (TaKaRa, Otsu, Japan) was used for each reaction. After an initial period of denaturation at 95°C for 10 min, 43 cycles of reactions were performed, each consisting of denaturation at 95°C for 1.5 min, annealing at 48°C for 1.5 min, and extension at 70°C for 2 min. PK114/K, a variant HPV16 clone provided by Dr Mattias Durst, was used as a positive control.

### Direct sequencing of PCR products

A partial L1 sequence was amplified by the primer L1C1. The amplified PCR products were purified, and automated sequencing was performed using an ABI Prism 3100 Genetic Analyser (Applied Biosystems, Foster City, CA, USA). The HPV type was determined based on an approximately 200 bases of L1 sequence and a search of the NCBI database (GenBank sequences; http://www.ncbi.nlm.nih.gov/blast/Blast.cgi) using Sequencing Analysis 3.3 software (The Perkin–Elmer Corporation, Norwalk, CT, USA).

### Statistics

Correlations in sensitivity between the ThinPrep slide and conventional Papanicolaou smear tests for the detection of cervical neoplasia and the concordance rate of each cytological method with the histological results were examined using McNemar's *χ*^2^ test. Correlations in sensitivity between the ThinPrep slide and HC II tests for the detection of cervical neoplasia were examined using the *χ*^2^ test. Correlations in the concordance rate of the HC II and PCR test results between the cases with CIN1 and those with CIN2, CIN3, and SCC were examined using Fisher's exact probability test. The HPV-positive rates were also examined using Fisher's exact probability test. *P*-values of 0.05 or less were considered to be statistically significant.

## RESULTS

Cytologic studies were performed in all 3000 cases. Twelve cases of glandular dysplasia, 11 cases of adenocarcinoma, five cases of adenocarcinoma *in situ* and one case of small cell carcinoma, all diagnosed cytologically, were excluded; the diagnostic results of the ThinPrep slide and conventional Papanicolaou smear specimens were then compared in the remaining 2971 cases (Table 1

**Table 1:** Diagnosis of ThinPrep slides and conventional smears

	**Conventional**					
**ThinPrep**	**Neg**	**BA**	**LGSIL**	**HGSIL**	**SCC**	**Total**
Neg	2579	9	18	0	0	2606
BA	3	16	4	2	0	25
LGSIL	17	3	151	21	0	192
HGSIL	2	0	12	106	4	124
SCC	0	0	0	0	24	24

Total	2601	28	185	129	28	2971

Neg=negative slide or smear; BA=benign atypia; LGSIL=low-grade squamous intraepithelial lesion; HGSIL=high-grade squamous intraepithelial lesion; SCC=squamous cell carcinoma.

). These 2971 cases consisted of patients with negative findings, benign atypia, low-grade squamous intraepithelial lesions, high-grade squamous intraepithelial lesions (HGSIL), or squamous cell carcinoma (SCC), based on their Papanicolaou test results. The concordance rate between the fluid-based Papanicolaou test results and the conventional Papanicolaou smear results was high (96.8%, 2876 out of 2971 cases). The percentage of cases with benign atypia did not differ significantly between the ThinPrep slide results (0.8%, 25 out of 2971 cases) and the conventional Papanicolaou smear results (0.9%, 28 out of 2971 cases, *P*=0.66).

Biopsy studies were performed in 557 of the 3000 cases. Based on the histological diagnosis, five cases of adenocarcinoma, five cases of adenocarcinoma *in situ*, three cases of condyloma acuminatum, two cases of adenosquamous cell carcinoma, and one case of small cell carcinoma were excluded from the 557 cases. A comparison between the cytological and histological results for the remaining 541 cases is shown in Tables 2

**Table 2:** Comparison between histological and conventional cytological diagnosis

	**Histological diagnosis**					
**Conventional**	**CC**	**CIN1**	**CIN2**	**CIN3**	**SCC**	**Total**
Neg	262	40	7	0	0	309
BA	8	2	2	1	0	13
LGSIL	41	40	14	2	0	97
HGSIL	12	12	31	43	1	99
SCC	0	0	0	6	16	22

Total	323	94	54	53	17	541

Neg=negative slide or smear; BA=benign atypia; LGSIL=low-grade squamous intraepithelial lesion; HGSIL=high-grade squamous intraepithelial lesion; CC=chronic cervicitis; CIN=cervical intraepithelial neoplasia; SCC=squamous cell carcinoma.

and 3

**Table 3:** Comparison between histological and ThinPrep cytological diagnosis

	**Histological diagnosis**					
**ThinPrep**	**CC**	**CIN1**	**CIN2**	**CIN3**	**SCC**	**Total**
Neg	264	42	7	2	0	315
BA	6	2	3	1	0	12
LGSIL	41	40	15	3	0	99
HGSIL	12	10	29	43	3	97
SCC	0	0	0	4	14	18

Total	323	94	54	53	17	541

Neg=negative slide or smear; BA=benign atypia; LGSIL=low-grade squamous intraepithelial lesion; HGSIL=high-grade squamous intraepithelial lesion; CC=chronic cervicitis; CIN=cervical intraepithelial neoplasia; SCC=squamous cell carcinoma.

. The absolute concordance rate between the cytological and histological results was 73.9% (400 out of 541 cases) for the fluid-based Papanicolaou test and 73.2% (396 of 541 cases) for the conventional Papanicolaou smear. This difference was not significant (*P*=0.12). The detection sensitivities for cervical intraepithelial neoplasia1 (CIN1), CIN2, CIN3, and SCC were 53.2, 81.5, 94.3, and 100.0%, respectively, for the ThinPrep slides, and 55.3, 83.3, 96.2, and 100.0%, respectively, for the conventional Papanicolaou smear specimens. These differences were not significant for any histological category (*P*>0.4). In the same manner, the CIN and SCC detection sensitivities also did not differ between the ThinPrep slides (73.9%, 161 out of 218 cases) and the conventional Papanicolaou smear specimens (75.7%, 165 out of 218 cases, *P*=0.22).

An HC II analysis was randomly performed in 477 of the 541 cases who underwent biopsy studies. Only cases positive for high-risk HPV were regarded as positive in this study; cases positive for only low-risk HPV were regarded as negative. The sensitivity of HC II for detecting CIN1, CIN2, CIN3, and SCC was 59.1% (55 out of 93 cases), 84.9% (45 out of 53 cases), 90.6% (48 out of 53 cases), and 100.0% (16 out of 16 cases), respectively. The overall CIN and SCC detection sensitivities did not differ significantly between the HC II (76.3%, 164 out of 215 cases) and the ThinPrep slide (73.9%, 161 out of 218 cases, *P*=0.56) test results.

Table 4

**Table 4:** Positive rate of HPV by PCR

		**Infection of HPV–(type of HPV)**				
**Histological diaganosis**	**Overall**	**HPV 16**	**HPV 52**	**Others**	**HPV 18**	**Others**
CIN1	37/51 (72.5%)	4 (7.8%)	2 (3.9%)	31 (60.8%)	1 (2.0%)	36 (70.6%)
CIN2	31/33 (93.9%)	8 (24.2%)	4 (12.1%)	19 (57.6%)	5 (15.2%)	26 (78.8%)
CIN3	35/41 (85.4%)	19 (46.3%)	9 (22.0%)	7 (17.1%)	1 (2.4%)	34 (82.9%)
SCC	12/14 (85.7%)	6 (42.9%)	1 (7.1%)	5 (35.7%)	2 (14.3%)	10 (71.4%)
AIS	2/3 (66.7%)	0 (0%)	0 (0%)	2 (66.7%)	1 (33.3%)	1 (33.3%)
AC	3/3 (100%)	0 (0%)	0 (0%)	3 (100%)	3 (100%)	0 (0%)
ASC	1/1 (100%)	0 (0%)	0 (0%)	1 (100%)	1 (100%)	0 (0%)

		*P<0.00001	**P<0.01		***P<0.001	

CIN=cervical intraepithelial neoplasia; SCC=squamous cell carcinoma; AIS=adenocarcinoma *in situ*; AC=adenocarcinoma; ASC=adenosquamous cell carcinoma.

* Fisher's exact probability test for positivity of HPV 16 *vs* others between CIN1–CIN2 and CIN3–SCC.

** Fisher's exact probability test for positivity of HPV 52 *vs* others between CIN1–CIN2 and CIN3–SCC.

*** Fisher's exact probability test for positivity of HPV 18 *vs* others between CIN1–SCC and AIS–AC.

summarises the HPV-positive rates as detected using direct sequencing of the PCR products. Fisher's exact probability test revealed that the frequency of cases with CIN3 or SCC, compared to those with CIN1 or CIN2, in the HPV 16-positive cases (67.6%, 25 out of 37 cases) or HPV 52-positive cases (62.5%, 10 out of 16 cases) was significantly higher than that of the other HPV types (19.4%, 12 out of 62 cases, *P* < 0.00001 for HPV 16, *P* < 0.01 for HPV 52). We also found that the frequency of cases with adenocarcionoma or adenocarcinoma *in situ*, compared to those with CIN or SCC, in the HPV 18-positive cases (30.8%, four out of 13 cases) was higher than that of the other HPV types (0.9%, one out of 107 cases, *P* < 0.001). HPV 18 was also detected in one case of the adenosquamous cell carcinoma.

The concordance rate between the HC II and PCR test results was 68.1% (32 out of 47 cases) in cases with CIN1, 88.4% (76 out of 86 cases) in cases with CIN2, CIN3, or SCC, and 81.2% (108 out of 133 cases) overall (Table 5

**Table 5:** Comparison between HC II and PCR for the detection of high-risk HPV DNA

	**CIN1**		**CIN2, CIN3, and SCC**	
	**HC II**		**HC II**	
	**(+)**	**(−)**	**(+)**	**(−)**
PCR (+)	20	13	72	4
(−)	2	12	6	4

CIN=cervical intraepithelial neoplasia; SCC=squamous cell carcinoma.

). The concordance rate between test results for cases with CIN2, CIN3, and SCC was higher than that for cases with CIN1 (*P*<0.01).

**Figure 1:**
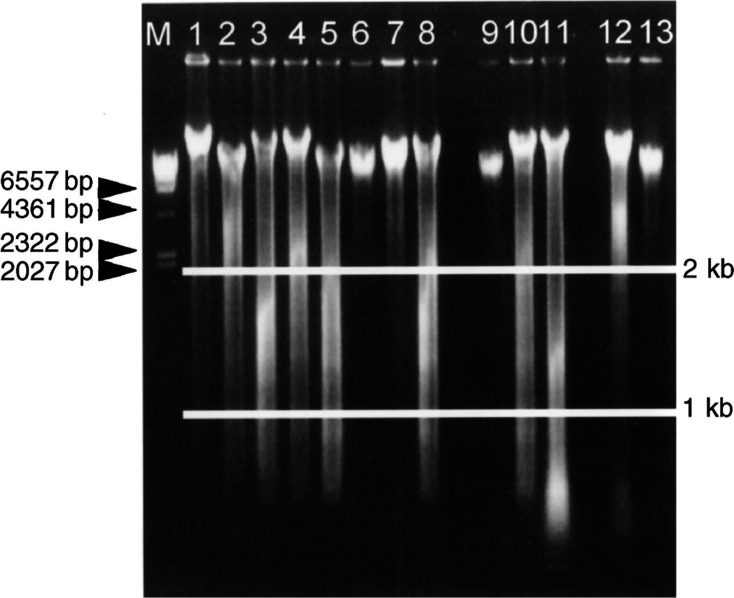
Estimating the quality of the extracted genomic DNA. Genomic DNA was electrophoresed on a 0.8% agarose gel. The quality of the extracted genomic DNA was classified into three categories. Genomic DNA samples 1, 2, 4, 6, 7, 9, 12, and 13 were classified as exhibiting grade 1 degradation. Genomic DNA samples 3, 5, 8, and 10 were classified as exhibiting grade 2 degradation. Genomic DNA sample 11 was classified as exhibiting grade 3 degradation. M shows the *λ*DNA/*Hind*III marker.

**Figure 2:**
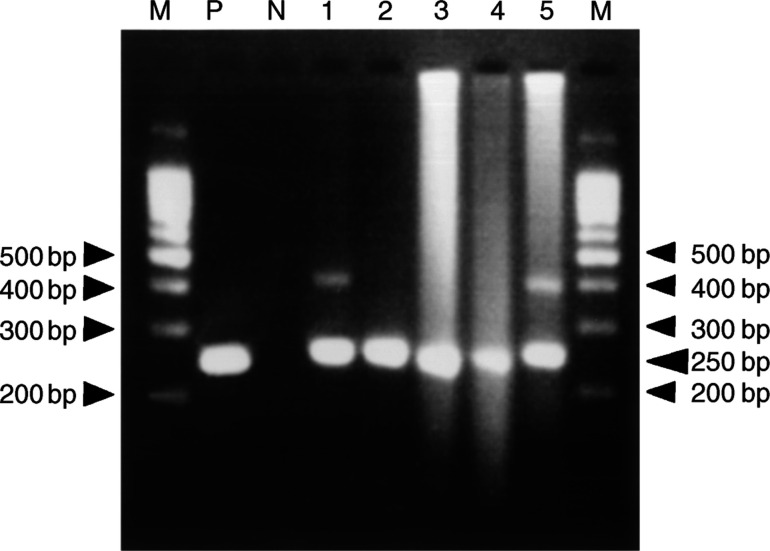
Detection of PCR products using electrophoresis. PCR products were electrophoresed on a 3% agarose gel. Lane 1, PCR products using genomic DNA with grade 1 degradation as a template; lane 2, PCR products using genomic DNA with grade 2 degradation as a template; lanes 3, 4, and 5, PCR products using genomic DNA with grade 3 degradation as a template. M shows the 100-bp DNA ladder marker. P shows the positive control. N shows the negative control.

While performing the PCR analyses, we found that some DNA samples extracted from the fluid-based specimens were degradaded when examined by electrophoresis. We analysed 170 samples in which the yield of the extracted DNA was more than 5 *μ*g in total. We then categorised the extracted DNA samples into grade 1, grade 2, or grade 3 degradation (Figure 1

). DNA samples containing fragments mainly larger than 2 kb were classified as grade 1 degradation. DNA samples containing 1–2 kb fragments were classified as grade 2 degradation. DNA samples containing fragments smaller than 1 kb were classified as grade 3 degradation. Grade 1, grade 2, and grade 3 degradation were seen in 96 cases (56%), 51 cases (30%), and 23 cases (14%), respectively. Although the positive bands for PCR products from a given genomic DNA with grade 1 or grade 2 degradation were clear, those from genomic DNA with grade 3 degradation were not because the degraded genomic DNA masked the PCR products. However, positive bands could be detected in all of the samples (Figure 2

). Consequently, PCR and direct sequencing was successfully performed in all the DNA samples, even after some of the samples had been stored for 12 months, although some DNA samples extracted from the fluid-based specimens had degraded.

## DISCUSSION

In this report, we performed the first large-scale study in Japan to examine the use of a new fluid-based Papanicolaou specimen collection system in 3000 Japanese women; the results were then compared to those of conventional smears. We also evaluated the use of residual fluid-based specimens after long-term storage in several molecular analyses, such as HPV screening by HC II, HPV detection by PCR, and HPV typing by sequencing.

The ThinPrep 2000 fluid-based, thin layer automated slide preparation system was designed to improve sample collection and preparation. Since fluid-based specimens can be stored at ambient temperatures for more than 1 year, this collection system is advantageous in that the Papanicolaou specimen can be re-examined several times using the residual specimen after the initial Papanicolaou test has been performed. In addition, this collection system makes it possible to perform follow-up studies without unduly inconveniencing the patient.

Several studies have reported that molecular analyses can be performed using fluid-based specimens and that these specimens enable the integrity of cellular DNA, RNA, and proteins to be retained (Lin WM *et al*, 2000b; Tarkowski *et al*, 2001). Therefore, molecular analyses can be performed using the residual specimen after a cytological diagnosis has been made, without the need to collect a second specimen from the patient. To our knowledge, however, the use of fluid-based Papanicolaou test specimens for HPV detection has not been reported in Japan.

This study of 3000 patients demonstrated that the quality of fluid-based ThinPrep slides is similar to that of conventional Papanicolaou smears, with regard to their use in screening and cervical neoplasia detection. Our results revealed a high concordance rate between the ThinPrep slide and conventional Papanicolaou smear test results. The CIN and SCC detection sensitivities and the percentage of cases with benign atypia did not differ between the two methods. However, some studies in Europe and America have reported an increased sensitivity for the detection of cervical neoplasia using the ThinPrep slide method (Awen *et al*, 1994; Linder and Zahniser, 1997; Roberts *et al*, 1997; Papillo *et al*, 1998). Other studies have reported that the rate of atypical squamous cells of undetermined significance decreased with the use of ThinPrep slides (Bolick and Hellman, 1998; Park *et al*, 2001). These differences are probably related to differences in cervical neoplasia screening programmes. In Japan, more than 5000 cytotechnologists are working in this field, so cytotechnologists can spend a longer time per case than in other countries. Although the two methods are similar in quality, we prefer the fluid-based Papanicolaou test because fluid-based specimens can be stored at ambient temperatures for long periods and can be used in other ways, such as in molecular analyses.

HC II was recently reported to be a useful kit for detecting HPV DNA (Clavel *et al*, 1998; Lin CT *et al*, 2000a; Castle *et al*, 2002). Thirteen types of high-risk HPV and five types of low-risk HPV can be detected using HC II. Ferenczy reported that the fluid-based cytologic system provides adequate material for hybrid capture tests (Ferenczy *et al*, 1996). Our results also showed that fluid-based specimens could be used in the HC II test. The sensitivity of HC II for detecting CIN and SCC was the same as that of the ThinPrep slides. The sensitivity of HC II for detecting HGSIL was also similar to previously reported values (73–93%) (Clavel *et al*, 1998; Kuhn *et al*, 2000; Lin CT *et al*, 2000a; Yamazaki *et al*, 2001). HC II may be a useful test for detecting cervical neoplasia in countries without cytologic screening systems. We prefer the Papanicolaou test, however, because more information, such as the grade of the dysplastic cells, can be obtained; the sensitivities of the Papanicolaou and HC II tests were similar.

We found that HPV 16 and HPV 52 were the predominant HPV types found in CIN3 and SCC, based on the direct sequencing of PCR products; consequently, CIN cases that are positive for these HPV types should be more strictly followed up than cases positive for other HPV types. Yoshikawa *et al* (1999) reported that HPV types 16, 18, 31, 33, 51, 52, and 58 were predominantly detected in HGSIL. Sasagawa *et al*. (2001) reported that HPV types 16, 31, 33, 35, 45, 51, 52, 56, and 58 were associated with HGSIL, while HPV types 16, 18, 31, 51, 52, and 58 were associated with SCC; thus infection with HPV type 16, 18, 31, 51, 52, or 58 is considered to be associated with a high-risk of CIN and SCC in Japanese women. Our results also revealed that HPV 18 was predominantly detected in adenocarcinoma, as previously reported (Lizano *et al*, 1997; Shyu *et al*, 2001). We previously reported that the incidence of multiple HPV coinfection was 38.3% in cervical dysplasia (Nakagawa *et al*, 2002). The significance of multiple HPV coinfection is controversial; therefore, our strategy for elucidating cells with multiple HPV coinfection to be performed in the future experiments may be useful.

The concordance rate between HC II and PCR test results has been reported to be high (Clavel *et al*, 1998; Peyton *et al*, 1998; Terry *et al*, 2001), but one study reported a rate of only 83% (Yamazaki *et al*, 2001). Our concordance rate (81.2%) was similar to that in the report by Yamazaki *et al*. Our study also revealed that the concordance rate between test results in CIN1 cases (68.1%) was lower than that in more progressive lesions (88.4%). The cause of the lower concordance rate among CIN1 cases is probably related to a low copy number of the HPV genome and a higher PCR sensitivity for the detection of HPV. In addition, HPV types that were not included in Probe B cocktail of HC II or unknown types of HPV can be detected using the PCR method.

In conclusion, the fluid-based Papanicolaou test produces specimens of the same quality as those produced using conventional Papanicolaou smears. ThinPrep sample vials can be stored at ambient temperature for periods of more than 1 year; furthermore, the detection of HPV DNA can be performed using residual specimens after cytological or biopsy studies have been completed. Moreover, a follow-up protocol consisting of concurrent cytology and HPV-DNA detection can be performed in patients with a high risk of CIN or SCC. This collection system should enable significant advances in molecular epidemiology and is useful for identifying high-risk cases of cervical cancer.
